# Argon Plasma Exposure Augments Costimulatory Ligands and Cytokine Release in Human Monocyte-Derived Dendritic Cells

**DOI:** 10.3390/ijms22073790

**Published:** 2021-04-06

**Authors:** Sander Bekeschus, Dorothee Meyer, Kevin Arlt, Thomas von Woedtke, Lea Miebach, Eric Freund, Ramona Clemen

**Affiliations:** 1 The Centre for Innovation Competence (ZIK) Plasmatis, Leibniz Institute for Plasma Science and Technology (INP), 17489 Greifswald, Germany; dorothee.j.meyer@gmail.com (D.M.); kevin.arlt@inp-greifswald.de (K.A.); woedtke@inp-greifswald.de (T.v.W.); lea.miebach@inp-greifswald.de (L.M.); eric.freund@inp-greifswald.de (E.F.); ramona.clemen@inp-greifswald.de (R.C.); 2 Institute of Hygiene and Environmental Medicine, Greifswald University Medical Center, 17475 Greifswald, Germany; 3 Department of General, Visceral, Thoracic, and Vascular Surgery, Greifswald University Medical Center, 17475 Greifswald, Germany

**Keywords:** CAP, cancer, cold atmospheric pressure plasma, hydrogen peroxide, hypochlorous acid, moDCs, peroxynitrite, reactive oxygen and nitrogen species, RNS, ROS

## Abstract

Cold physical plasma is a partially ionized gas expelling many reactive oxygen and nitrogen species (ROS/RNS). Several plasma devices have been licensed for medical use in dermatology, and recent experimental studies suggest their putative role in cancer treatment. In cancer therapies with an immunological dimension, successful antigen presentation and inflammation modulation is a key hallmark to elicit antitumor immunity. Dendritic cells (DCs) are critical for this task. However, the inflammatory consequences of DCs following plasma exposure are unknown. To this end, human monocyte-derived DCs (moDCs) were expanded from isolated human primary monocytes; exposed to plasma; and their metabolic activity, surface marker expression, and cytokine profiles were analyzed. As controls, hydrogen peroxide, hypochlorous acid, and peroxynitrite were used. Among all types of ROS/RNS-mediated treatments, plasma exposure exerted the most notable increase of activation markers at 24 h such as CD25, CD40, and CD83 known to be crucial for T cell costimulation. Moreover, the treatments increased interleukin (IL)-1α, IL-6, and IL-23. Altogether, this study suggests plasma treatment augmenting costimulatory ligand and cytokine expression in human moDCs, which might exert beneficial effects in the tumor microenvironment.

## 1. Introduction

The resolution of many diseases is controlled by precise modulation of inflammation [1,2,3]. Pro-inflammatory responses help to promote pathogen clearance and antigen presentation to engage adaptive immunity, while anti-inflammatory responses often counterbalance preceding inflammation, which—if left unchecked—lead to tissue damage and cellular dysfunction. Cells of the innate immune system critically modulate inflammatory responses and provide the link between a pathological condition, e.g., infection or cancer and adaptive immune responses that can, for instance, specifically target infected or malignant cells. Especially, dendritic cells (DCs) are the most prominent example of providing activating antigens for T cell stimulation, which is why these cells are a member of the professional antigen-presenting cells (APCs) family [4].

Two major DC subsets exist: conventional DCs (cDCs) and plasmacytoid DCs (pDCs). The former are characterized by toll-like receptor (TLR) 2 and 4 expression and can be further divided into cDC-1, which are more abundant and provide major stimulus for T cells, and for anticancer immunity, and the less abundant cDC-2, which are critical in targeting infection, e.g., in wounds [5]. The latter (pDCs) mainly express TLR7, TLR8, and TLR9, and are also critical in antitumor effects due to their direct cytotoxic activity towards cancer cells, such as melanoma [6]. DCs can be differentiated from human monocytes via chemokine/cytokine stimulation that leads to the upregulation of several surface receptors crucial for antigen presentation to T cells, such as major histocompatibility complex class II (MHC-II or HLA-DR), and cluster of differentiation (CD) 40, CD80, CD83, and CD86, along with changes in their metabolic profiles [7]. Antigen presentation is done after maturation and migration in a CCR7-dependent manner to secondary lymphatic organs such as the lymph node or spleen, which are patrolled by naïve T cells searching for their cognate antigen [8]. Reactive oxygen and nitrogen species (ROS/RNS) also play vital roles in the biology of DCs as their intracellular levels correlate with the priming, function, and development of these cells, with implications in inflammatory responses [9].

ROS/RNS release is the prime hallmark of cold physical plasmas. This accredited technology has been established in many dermatological centers with the main focus on wound management in the last decade [10] and is based on evidence from several clinical trials [11,12,13]. Many in vivo models have supported the claim of physical plasma-induced healing being also promoted in sterile wounds, i.e., in the absence of infection [14,15,16]. Moreover, recent evidence found a role of physical plasma treatment in oncology [17]. Cancer patients benefited from this treatment during palliation [18], and several syngeneic animal models suggested an involvement of antitumor immunity unleashed by plasma-induced tumor cell killing and subsequent putative transport of tumor antigens by DCs promoting T cell activation [18,19,20,21]. The patients’ results also suggest the role of the immune system [22], with DCs being among the key cells takiing up antigens from inactivated tumor cells. Ultimately, this suggests physical plasma-derived ROS/RNS, which are plentiful and diverse [22], directly affect ROS/RNS sensing and redox signaling and ultimately contributing to biologically relevant consequences [23].

While the tumor-toxic activity of physical plasma treatment has been shown numerous times [24], and it is clear that DCs are propagating antitumor immunity in the cancer context [25], it has not been investigated so far how plasma exposure affects DCs alone that—in an in vivo setting—undoubtedly are present in the tumor microenvironment (TME) [26]. To this end, we investigated DC activity, surface marker expression, and cytokine release in human-monocyte-derived DCs (moDCs) in vitro following physical plasma exposure and compared responses to treatments with single ROS/RNS and lipopolysaccharide (LPS). All treatments had an impact on DCs, while plasma treatment elicited notable changes potentially beneficial for their function.

## 2. Results

### 2.1. Toxicity towards Argon Plasma Treatment and ROS/RNS

**Figure 1:**
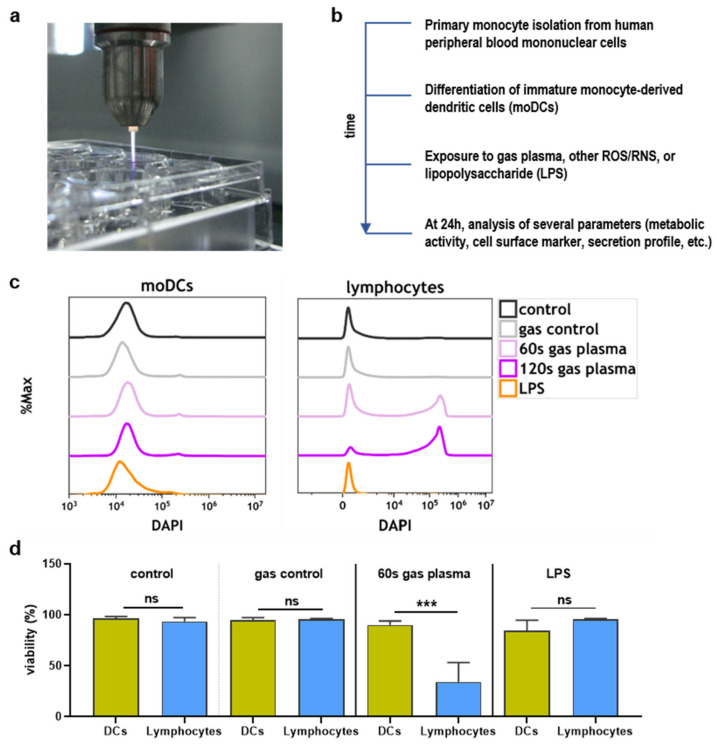
Study protocol and toxicity comparison. (**a**) Image of argon plasma treatment of cells in 24-well plates; (**b**) scheme of study protocol; (**c**,**d**) overlay 4′,6-diamidino-2-phenylindole (DAPI) histograms of monocyte-derived DCs (moDCs) and lymphocytes according to the indicated treatments (**c**) and quantification of the percentage of viable moDCs and lymphocytes (**d**) treated together in a single well. Data are mean and standard error of three experiments, and statistical analysis was performed using t-test with *p* < 0.001 (***) differing significantly or non-significantly (ns).

In this study, the kINPen argon plasma jet was used (Figure 1a) for the treatment of monocyte-derived dendritic cells (moDCs). Analysis was conducted 24 h later (Figure 1b). To estimate the sensitivity of moDCs compared to lymphocytes, cell suspensions were treated together, and the number of dead cells was analyzed for each population separately (Figure 1c). It was found that lymphocytes were markedly more sensitive to argon plasma-induced cytotoxic effects compared to the moDCs (Figure 1d).

**Figure 2:**
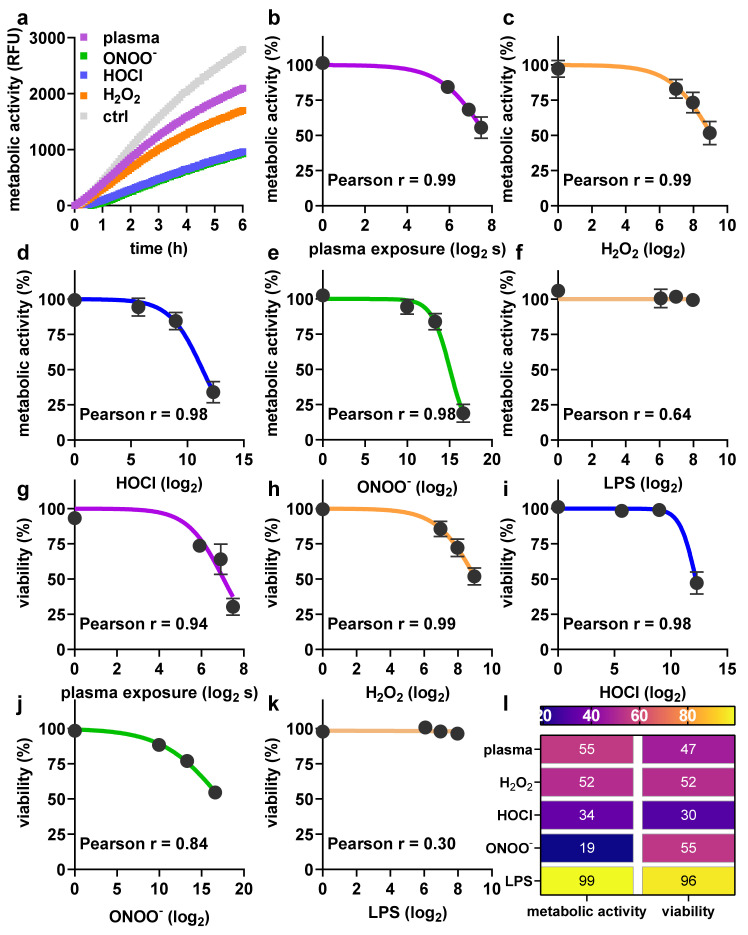
Argon plasma treatment and reactive oxygen and nitrogen species (ROS/RNS) have dose-dependent toxicity profiles. (**a**) Kinetic metabolic activity of moDCs treated as indicated over 6 h; (**b**–**f**) metabolic activity in response to several argon plasma treatments times or concentrations of ROS/RNS or LPS at 24 h; (**g**–**k**) viability in response to several argon plasma treatment times or concentrations of ROS/RNS or lipopolysaccharide (LPS) at 24 h; and (**l**) heatmap for comparison between reduction in metabolic activity and viability. Data are mean and standard error of 3–6 different donors.

Next, the metabolic activity and viability of moDCs following exposure to argon plasma or ROS/RNS were investigated. Pronounced differences were observed for metabolic activity when assayed kinetically after exposure to pilot amounts of ROS/RNS or plasma treatment (Figure 2a), demonstrating the assay sensing differences in metabolic activity in stress inactivated cells. Subsequently, metabolic activity endpoint assays were conducted 24 h after exposure to different argon plasma treatment times (Figure 2b) or concentrations of hydrogen peroxide (H_2_O_2_, Figure 2c), hypochlorous acid (HOCl, Figure 2d), peroxynitrite (ONOO^-^, Figure 2e), and lipopolysaccharide (LPS, Figure 2f). Except for LPS, which is known to be non-toxic at lower concentrations, all agents showed a treatment time-dependent reduction in metabolic activity. These data were in agreement with the results obtained via flow cytometry assaying cell viability (Figure 2g–k), as illustrated by the heatmap (Figure 2l).

### 2.2. Surface Marker Expression and Cytokine Release after Plasma or ROS/RNS Exposure

**Figure 3:**
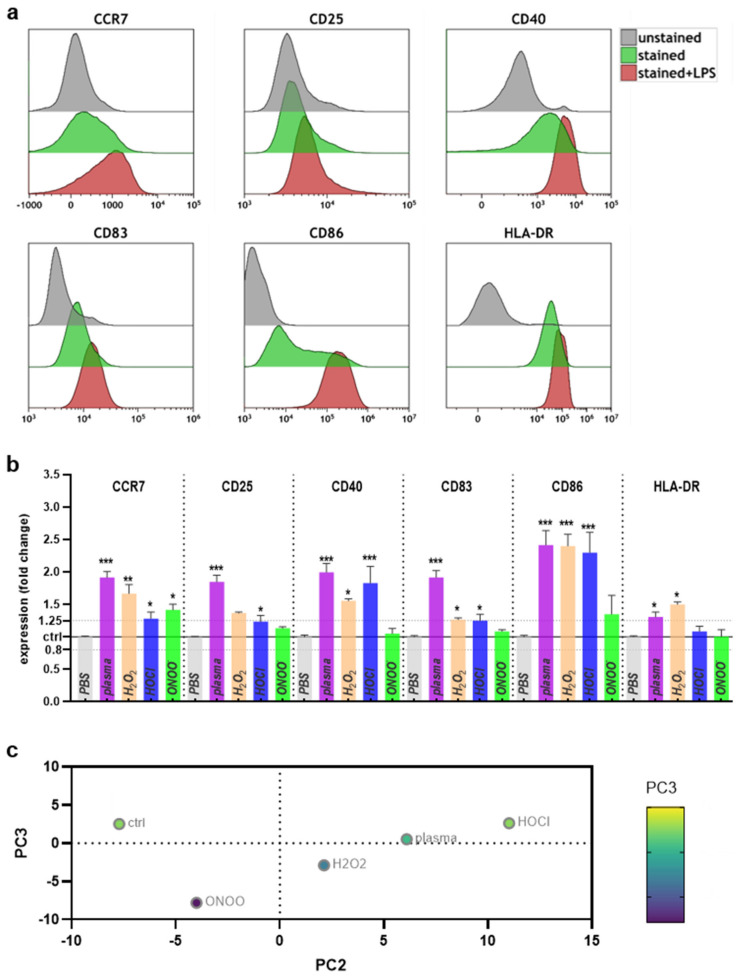
Argon plasma treatment and ROS/RNS modulate the surface marker expression profiles. (**a**) overlay histograms of several cell surface markers of unstained, stained, stained, and LPS-pulsed human moDCs; (**b**) quantification and fold-change differences in human moDCs treated as indicated and analyzed 24 h later; (**c**) data summary using principal-component analysis. Data are mean and standard error of 3–6 different donors. Statistical analysis was done using one-way analysis of variances with *p* < 0.05 (*), *p* < 0.01 (**), and *p* < 0.001 (***).

Next, the surface marker expression levels on moDCs were investigated. Pilot experiments using unstained, stained, and LPS-pulsed, and stained cells confirmed the known upregulation of several costimulatory molecules after activation, including CCR7, CD25, CD40, CD83, CD86, and HLA-DR (Figure 3a).

Next, moDCs were exposed to partially toxic argon plasma or ROS/RNS conditions, and the surface marker expression was investigated by multicolor flow cytometry and normalized to that of cells receiving vehicle only. Shown are also the boundary lines for ±1.25 differences in fold-change, and a significant downregulation was not observed for any of the markers or conditions (Figure 3b). Argon plasma treatment led to a pronounced increase of expression across all markers investigated. Exposure to H_2_O_2_ produced similar but less pronounced results. The same findings were made for HOCl, except for lack of significant increase of CD25 and HLA-DR. ONOO^-^ exposure resulted in the most minor changes across the ROS/RNS treatments, with a significant increase observed for CCR7 only. The surface marker expression values were subsequently fed into a principal component analysis, showing that argon plasma treatment was more similar to H_2_O_2_ and HOCl than ONOO^-^ (Figure 3c). Altogether, the results suggest an increase of costimulatory surface marker expression in moDCs, which was pronounced for argon plasma treatment and observed for the other ROS/RNS regimens as well.

**Figure 4:**
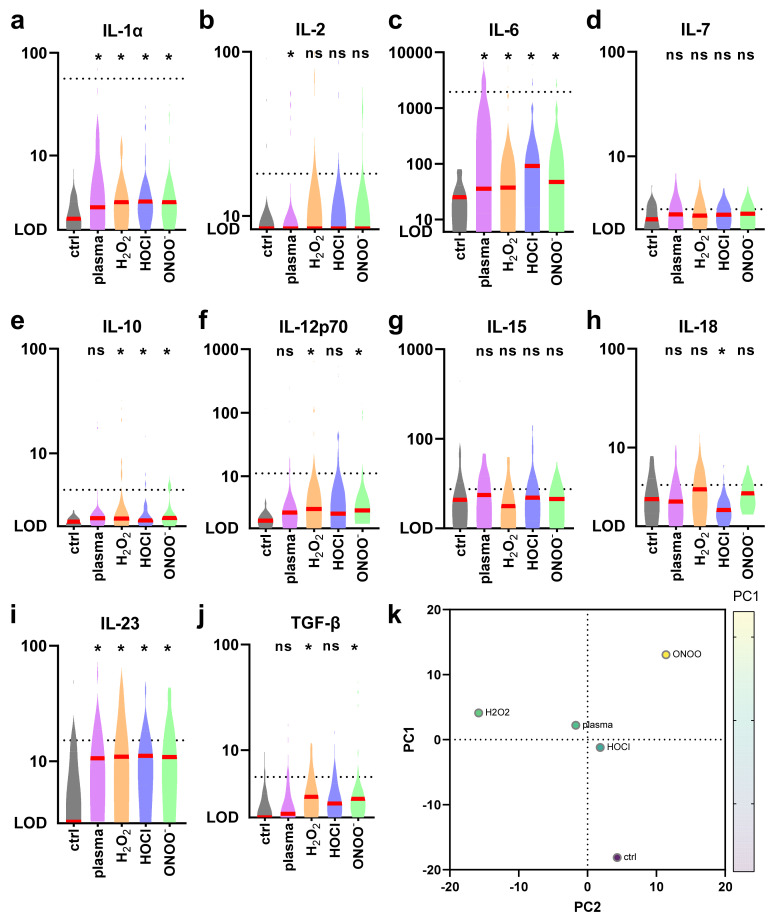
Argon plasma treatment and ROS/RNS modulate the cytokine release profiles. Cells were treated as indicated, supernatants were collected 24 h later, and absolute cytokine concentrations of 10 analytes (**a**–**j**) were assessed. All data were also related to each other using principal component analysis (**k**). Data are violin plots and median (red lines) of 3–6 different donors. Statistical analysis was done using one-way analysis of variances with *p* < 0.05 (*). Dashed lines show values of LPS-positive controls of moDCs included for the analytes.

Next, the secretory profiles of the treatment conditions were examined for 10 different cytokines (Figure 4a–j). Argon plasma treatment led to significantly elevated levels of IL-1α, IL-2, IL-6, and IL-23. For H_2_O_2_, levels significantly increased from that of controls samples for IL-1α, IL-6, IL-10, IL-12p70, IL-23, and TGF-β. In the case of HOCl exposure, significant differences were found for IL-1α, IL-6, IL-10, IL-18, and IL-23. For ONOO^-^ treatments, significantly higher concentrations were found for IL-1α, IL-6, IL-10, IL-12p70, IL-23, and TGF-β. Despite the differences being significant, the amplitude of differences were subtle, overall. Consistent changes across all ROS/RNS treatments were found for IL-1α, IL-6, and IL-23. All data were fed into a principal component analysis, revealing the argon plasma condition being in between H_2_O_2_ and HOCl and more apart from ONOO^−^ conditions, which mimicked the results obtained for the surface marker expression. These data provided evidence of argon plasma treatment mildly increasing the inflammatory cytokine profile in human moDCs.

## 3. Discussion

Dendritic cells (DCs) are critical for eliciting antitumor immunity and present in the tumor microenvironment (TME) that is envisaged to be targeted with cold physical plasma. While antitumor effects of this technology have been shown in many experimental models, the effects of argon plasma treatment on DCs have not been elucidated so far. This was the current study’s aim.

We found that monocyte-derived DCs (moDCs) were much more resistant to argon plasma-induced cytotoxic effects compared to lymphocytes. This mirrors previous findings comparing lymphocyte survival to that of undifferentiated monocytes after argon plasma exposure and showing a survival advantage of the latter in both primary human cells as well as cell lines [27,28]. Mechanistically, previous studies suggested an association between the enhanced oxidative stress resistance of myeloid progenitor cells (stem-like cells) and increased intracellular expression of catalase, glutathione peroxidase, and manganese superoxide dismutase [29]. Vice versa, it has been shown that transduction of lymphocytes with catalase increased their viability in response to ROS treatment [30]. On the transcriptional level, FoxO was suggested to be associated with oxidative-stress resistance in hematopoietic cells [31]. In general, it is understood that monocytes and lineages deriving from them (e.g., DCs and macrophages) are less affected by ROS-induced cell death since these cells are capable of producing them themselves via activation of NADPH oxidases and myeloperoxidase at the cell membrane or in phagosomes, for instance [32]. Human moDCs, for instance, were previously found to generate millimolar concentrations of ROS in their phagosomes per second [33]. This, and the enhanced transcriptional regulation of redox-related genes in moDCs [34], underlines their enhanced oxidative stress resistance, which is greater in cDCs than pDCs [35].

DCs are present in the TME, and their role is to elicit T cell responses in secondary lymphatic organs via the presentation of tumor antigen [36]. In this process, DCs become activated and upregulate the surface expression of several costimulatory ligands, such as CD40, CD83, CD86, and MHC-II [37,38]. In our study, argon plasma and the exogenous addition of single ROS/RNS all significantly increased CD40, CD83, and CD86, which agrees with a previous study using H_2_O_2_ [35]. It should be noted that industrially produced H_2_O_2_ contains stabilizers, which might cause more individual effects than plasma and H_2_O_2_ derived from it. In professional antigen-presenting cells, CD40 ligation through CD154 activates DCs [39]. CD83 and CD86 are critical in providing costimulation for T cell activation, and especially elevated CD83 expression demarcates DC maturation [40]. The slight but significant increase of MHC-II in our study with argon plasma or H_2_O_2_ exposure underlines this notion, as MHC-II is upregulated in activated DCs, and ensures enhanced antigen presentation to T cells [41]. While many reports found H_2_O_2_ to be an important product for the physical plasma effects observed [42,43,44,45], other short-lived species and precursors might also be in place for reaction with biomolecules [46,47,48,49]. Another report also found an upregulation of TLR2 and TLR4 in response to H_2_O_2_ treatment [50], targets that were not investigated in our work. Hence, although functional T cell stimulation assays are lacking, our data suggest that especially argon plasma treatment and also ROS/RNS exposure, in general, may foster moDC activation as seen by their increased expression of costimulatory molecules. The effect of argon plasma treatment also was stronger than that of, e.g., H_2_O_2_ treatment alone. This might be due to the physical plasma treatment being a multimodality regimen generating a multitude of different ROS/RNS as well as other physical effects such as mild UV radiation and electric fields that may support the action of the plasma-derived ROS/RNS on the cells [51,52,53,54].

The altered cytokine secretion profiles support this. While the literature on moDC H_2_O_2_, HOCl, ONOO^-^ exposure, and multi-cytokine secretion of moDCs is scarce and for physical plasma treatment absent, there is ample knowledge on the role of the cytokines investigated in DC biology. IL-1α showed a slight but consistent increase with all treatments, and the cytokine can generate inflammatory DCs in an autocrine fashion [55]. IL-6 was also increased across all conditions, and its secretion has—similar to many other cytokines—pleiotropic effects. For instance, DC-derived IL-6 was found to promote colon cancer metastasis [56], drive TH17 differentiation in concert with IL-1 and TGF-β, and foster macrophage differentiation from monocytes [57]. Along similar lines, DC-derived IL-23, which was also found to be increased, is associated with inflammatory bowel disease [58], osteosarcoma progression [59], and autoinflammation and autoimmunity in general [60]. Nevertheless, it should be mentioned that the absolute changes in concentrations across all 10 cytokines investigated were very moderate, especially in the light of hundred-fold-changes observed in literature with activating agents such as TLR-agonists, Poly I:C, Interferon β, and TNF-α [61]. Therefore, a strong effect of the argon plasma or ROS/RNS induced cytokine changes found in our study might not be expectable. Notwithstanding the relatively small changes identified in our study, they served to consistently separate the different ROS/RNS treatments in principal component analysis in both surface marker expression and cytokine secretion profiles.

## 4. Materials and Methods

### 4.1. Dendritic Cells

Human PBMCs were isolated from donor blood from buffy coats dedicated for research purposes and obtained at the Institute of Transfusion Medicine (Greifswald University Medical Center, Greifswald, Germany) based on a density-gradient protocol as described before [62]. Subsequently, CD14^+^ monocytes were separated using magnetic isolation (BioLegend, Amsterdam, The Netherlands), and viable cell counts and purity were assessed using an attune Nxt flow cytometer (Applied Biosystems, Darmstadt, Germany). In each well of a 24-well plate (Eppendorf, Hamburg, Germany), 2 × 10^5^ cells were seeded in 500 µL of fully supplemented cell culture medium. The cell culture medium was Roswell-Park Memorial Institute (RPMI) 1640 medium supplemented with 10% fetal bovine serum, 5% glutamine, 0.1 mg/L penicillin, and 100 U/L streptomycin (all Sigma-Aldrich, Taufkirchen, Germany). Maturation was induced using human granulocyte-macrophage stimulating factor (GM-CSF, 800IU; PeproTech, Hamburg, Germany) and human interleukin (IL) 4 (IL-4, 500IU; PeproTech, Hamburg, Germany) to retrieve immature human monocytes-derived dendritic cells.

### 4.2. Argon Plasma Treatment and ROS Exposure

MoDCs were exposed to either hydrogen peroxide (H_2_O_2_ in water with 0.5 ppm stannate-containing compounds and 1 ppm phosphorus-containing compounds as stabilizers, final concentrations were 500 µM, 250 µM, and 125 µM; Sigma-Aldrich, Taufkirchen, Germany), hypochlorous acid (HOCl, final concentrations were 5000 µM, 500 µM, and 50 µM; Sigma-Aldrich, Taufkirchen, Germany), or peroxynitrite (ONOO^−^, final concentrations were 10,000 µM, 1000 µM, and 100 µM; Sigma-Aldrich, Taufkirchen, Germany) at different concentrations to obtain dose-response toxicity relationships. Alternatively, cells were left untreated, exposed to argon gas alone (2 standard liters per minute; Air Liquide, Hamburg, Germany), or exposed to argon plasma at different treatment times (60 s, 120 s, and 180 s). The atmospheric pressure argon plasma jet kINPen (neoplas, Greifswald, Germany) was used. The jet and its physical properties have been reviewed recently [63], with the operation frequency being 1 MHz and the plasma-dissipated power and input power being about 1 W and 20 W, respectively. The distance between the nozzle of the head and the liquid was 15 mm. The evaporation was compensated for by adding a pre-determined amount of double-distilled water after argon plasma treatment was finished to restore isosmotic conditions for the cells. Significant changes in the temperature of the liquid were not observed and were about 5 °C in increase from 20 °C [64], while the medium in the incubator was 37 °C. As positive control, lipopolysaccharide (LPS, final concentrations were 250 ng/mL, 125 ng/mL, and 67 ng/mL; Sigma-Aldrich, Taufkirchen, Germany) was used.

### 4.3. Flow Cytometry

After incubation for 24 h, supernatants and cells were collected, and cells were washed. For analyzing the cells’ viability, 4′,6-diamidino-2-phenylindole (DAPI, 1 µM; BioLegend, Amsterdam, The Netherlands) and CD11c conjugated to phycoerythrin (PE) cyanine (Cy) 7 (clone S-HCL-3; BioLegend, Amsterdam, The Netherlands) were added and incubated for 15 min at room temperature in the dark. Subsequently, cells were washed, resuspended in running buffer (Miltenyi Biotec, Bergisch-Gladbach, Germany), and cell data were acquired on a Gallios flow cytometer (Beckman-Coulter, Krefeld, Germany) equipped with an autosampler for FACS tubes. For surface marker analysis, cells were incubated with antibodies (Table 1), washed, and data were acquired on a CytoFLEX LX flow cytometer (Beckman-Coulter, Krefeld, Germany) equipped with an autosampler for 96-well plates. Data analysis was performed using Kaluza 2.1.1 software (Beckman-Coulter, Krefeld, Germany).

### 4.4. Supernatant Analysis

**Figure A1:**
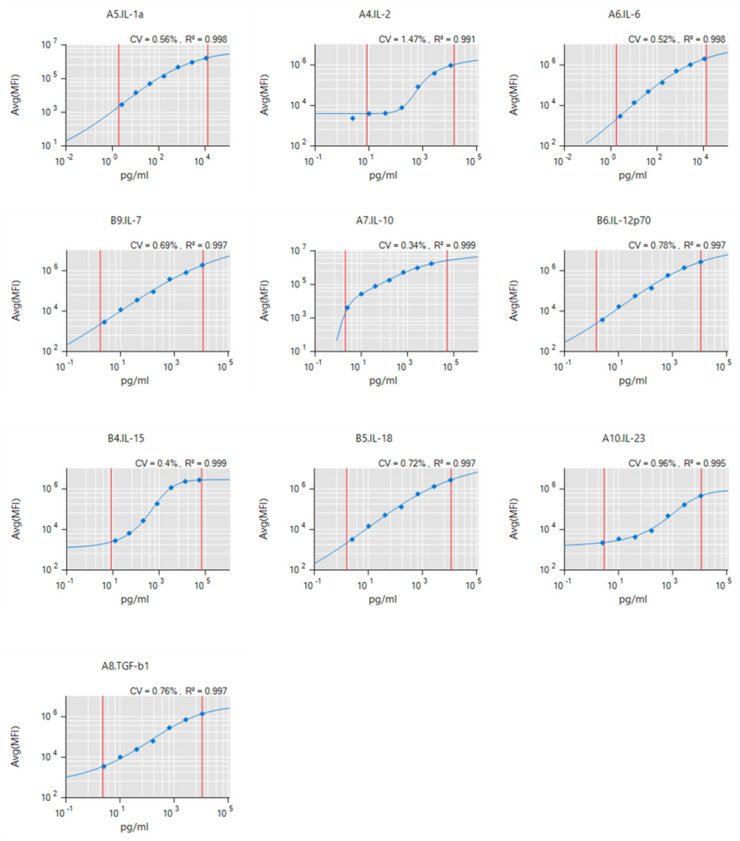
Standard curves from the multiplex cytokine analysis for each analyte across a range of concentrations shown together with the corresponding average mean fluorescence intensities (Avg(MFI)). All curve fittings had an *R*^2^ of greater 0.99, providing great accuracy for the quantification of absolute cytokine levels in moDC culture supernatants.

To assess metabolic activity 24 h after treatment of moDCs, 100 µM of resazurin (Alfa Aesar, Kandel, Germany) resolved in fully supplemented cell culture medium was added to each well. After 4 h of incubation at 37 °C in the incubator, the plate was read on a multimode plate reader (F200; Tecan, Männedorf, Switzerland) at λ_ex_ 535 nm and λ_em_ 590 nm. In some experiments, kinetic readings were performed on the device by preheating it up to 37 °C for 1 h, adding additional double-distilled water to the outer rim of the Eppendorf 24-well plate as evaporation shield protection, and continuously supplying 5% CO_2_ to the plate reader using the Gas Control Module (GCM; Tecan, Männedorf, Switzerland). For analyzing the concentration of cytokines in cell culture supernatants, the LEGENDplex (BioLegend, Amsterdam, The Netherlands) multiplex bead technology was used as recently described [65]. Briefly, supernatants were collected in 96-well plates and stored at −20 °C for longitudinal analysis. After thawing, supernatants were mixed with capture beads and antibodies, washed, and analyzed using a CytoFLEX S (Beckman-Coulter, Krefeld, Germany) flow cytometry equipped with an autosampler for 96-well plates. Absolute concentrations were calculated against a 5 log fit from a 7-seven serial dilution series (Figure A1) using LEGENDplex software (BioLegend, Amsterdam, The Netherlands).

### 4.5. Statistical Analysis

Statistical analysis was performed using prism 9.02 (GraphPad Software, San Diego, CA, USA). For comparison between two groups, t-test was used. Regressions were calculated from log-2 transformed data and non-linear fit; Pearson’s r was calculated to obtain the goodness of fit. For comparing more than two groups, one-way analysis of variances was performed. For principal component analysis (PCA), data were standardized and PCs were selected based on parallel analysis using Monte Carlo simulations on random data of equal dimensions to the input data and subsequent calculation of eigenvalues for all resulting PCs.

## Figures and Tables

**Table 1:** Antibodies used in this study.

Target	Clone	Conjugate
CD11c	S-HCL-3	PE-Cy7
CD25	BC96	Alexa Fluor 488
CD40	5C3	APC
CD83	HB15	PerCP-Cy5.5
CD86	IT2.2	PE
CD197	GO43H7	Brilliant Violet 785
HLA-DR	APC-Cy7	APC-Cy7

## Data Availability

The data presented in this study are available on request from the corresponding author.

## Appendix A

The determination of absolute levels of cytokines was done against a fit of a seven-fold dilution series of each of the analytes (Figure A1). The goodness of fit for each of the analytes across this dilution series was always higher than *R*^2^ > 0.99, providing the basis for accurate quantification for each sample and analyte. The coefficient of variation (CV) of each of the dilution series was always smaller than 1 (except for IL2 due to the last dilution step).
